# Gut Microbiome Diversity in European Honeybees (*Apis mellifera* L.) from La Union, Northern Luzon, Philippines

**DOI:** 10.3390/insects16020112

**Published:** 2025-01-23

**Authors:** Diana Castillo, Evaristo Abella, Chainarong Sinpoo, Patcharin Phokasem, Thunyarat Chantaphanwattana, Rujipas Yongsawas, Cleofas Cervancia, Jessica Baroga-Barbecho, Korrawat Attasopa, Nuttapol Noirungsee, Terd Disayathanoowat

**Affiliations:** 1 Department of Biology, Faculty of Science, Chiang Mai University, Chiang Mai 50200, Thailand; dccastillo@clsu.edu.ph (D.C.); chainarong_s@cmu.ac.th (C.S.); patcharin.ph@cmu.ac.th (P.P.); thunyarat.chan@gmail.com (T.C.); r.yongsawas@gmail.com (R.Y.); 2 Department of Biological Sciences, College of Science, Central Luzon State University, Science City of Muñoz 3120, Nueva Ecija, Philippines; eaabella@clsu.edu.ph; 3 Research Center of Deep Technology in Beekeeping and Bee Products for Sustainable Development Goals (SMART BEE SDGs), Chiang Mai University, Chiang Mai 50200, Thailand; korrawat.a@cmu.ac.th; 4 Office of Research Administration, Chiang Mai University, Chiang Mai 50200, Thailand; 5 Bee Program, University of the Philippines–Los Baños, Los Baños 4031, Laguna, Philippines; cleofascervancia@gmail.com (C.C.); jbbaroga@up.edu.ph (J.B.-B.); 6 Department of Entomology and Plant Pathology, Faculty of Agriculture, Chiang Mai University, Chiang Mai 50200, Thailand; 7 Center of Excellence in Microbial Diversity and Sustainable Utilization, Faculty of Science, Chiang Mai University, Chiang Mai 50200, Thailand

**Keywords:** bacterial microbiota, honeybee functional microbiota, Illumina MiSeq, insects, metagenomics, fungal microbiota, next-generation sequencing, Philippines

## Abstract

Insects often rely on symbiotic bacteria and fungi for various physiological processes, developmental stages, and defenses against parasites and diseases. Despite their significance, the associations between bacterial and fungal symbionts in *Apis mellifera* are not well studied, particularly in the Philippines. In this study, we collected *A. mellifera* from two different sites in the Municipality of Bacnotan, La Union, Philippines. A gut microbiome analysis was conducted using next-generation sequencing with the Illumina MiSeq platform. Bacterial and fungal community compositions were assessed using 16S rRNA and ITS gene sequences, respectively. Our findings confirm that adult worker bees of *A. mellifera* from the two locations possess distinct but comparably proportioned bacterial and fungal microbiomes. Key bacterial symbionts, including *Lactobacillus*, *Bombilactobacillus*, *Bifidobacterium*, *Gilliamella*, *Snodgrassella*, and *Frischella*, were identified. The fungal community was dominated by the yeasts *Zygosaccharomyces* and *Priceomyces*. Using the ENZYME nomenclature database and PICRUSt2 software version 2.5.2, a predicted functional enzyme analysis revealed the presence of β-glucosidase, catalase, glucose-6-phosphate dehydrogenase, glutathione transferase, and superoxide dismutase, which are involved in host defense, carbohydrate metabolism, and energy support. Additionally, we identified notable bacterial enzymes, including acetyl-CoA carboxylase and AMPs nucleosidase. Interestingly, the key bee symbionts were observed to have a negative correlation with other microbiota. These results provide a detailed characterization of the gut microbiota associated with *A. mellifera* in the Philippines and lay a foundation for further metagenomic studies of microbiomes in native or indigenous bee species in the region.

## Simple Summary

The honeybee *Apis mellifera* plays a crucial role in pollination, supporting high-value crops, flowers, and field crops while contributing significantly to biodiversity by maintaining ecosystems that largely depend on honeybee pollination. Increasing attention has been given to the honeybee gut microbiota, as its microbial community is essential for host health. However, in the Philippines, the gut microbiome of *A. mellifera* remains underexplored, and its functional roles and microbial network interactions are poorly understood. This study reveals that the gut microbiome of *A. mellifera* in the Philippines is dominated by key bacterial symbionts, particularly *Lactobacillus*, and a fungal community primarily composed of *Zygosaccharomyces*. A predicted functional analysis highlighted enzymes involved in host defense, carbohydrate metabolism, and energy production. A network analysis further demonstrated negative associations between dominant bacteria and fungi with other micro-organisms. These findings provide valuable insights with potential applications in the apiculture industry, pollinator conservation, and food security.

## 1. Introduction

European honeybees (*Apis mellifera* L.) are ubiquitous floral visitors in natural environments, playing a vital role in pollinating diverse ecosystems, from lowlands to mountains, and they are essential for global food security [1,2]. Similar to the balanced and healthy human microbiome, bees have evolved to harbor a unique gut microbiome through symbiotic relationships with micro-organisms [3]. This gut microbiome significantly influences the well-being of animal life, including insects [4], and has been extensively studied for its critical roles in bee health and disease [5].

Honeybees possess a simple yet distinct and conserved gut microbiota [6,7,8]. The honeybee gut bacterial microbiome has been thoroughly investigated regarding taxonomy [9], function, and mechanisms [10,11,12,13], as well as its response to environmental conditions, including abiotic and biotic stress [14,15,16]. These mechanisms apply similarly when studying the fungal microbiome [17,18], viruses [19,20,21], and other pests and diseases [22]. Advanced molecular techniques have provided an overview of six primary bacterial taxa in the honeybee microbiota: α-, β-, γ-proteobacteria, Firmicutes, Bacteroidetes, and Actinobacteria [23]. Metagenomic studies on *A. mellifera* have revealed eight major bacterial species, including *Lactobacillus* and *Bifidobacterium* from the Firmicutes phylum, *Snodgrassella alvi* from Betaproteobacteria, *Gilliamella apicola*, and *Frischella perrara* from γ-proteobacteria, among others [9,10]. In the fungal community of honeybees, abundant organisms such as yeasts (*Starmerella*, *Metschnikowia*, *Zygosaccharomyces*, *Wickerhamomyces*, and *Candida*) and filamentous fungi (*Aspergillus*, *Penicillium*, *Cladosporium*, and *Moniliella*) have been identified [17,24,25,26,27].

The Philippines, part of tropical Asia, is uniquely suited for beekeeping due to its abundance of pollen and nectar resources and due to it being home to several native honeybee species. Five of the nine honeybee species worldwide are native to the Philippines: *Apis andreniformis*, *A. breviligula*, *A. cerana*, *A. dorsata*, and *A. nigrocincta* [28]. Despite the introduced *A. mellifera* experiencing colony declines [29], it remains a crucial pollinator for many crops critical for food security and sustainable economic growth [30,31]. *A. mellifera* is the most widely used species in commercial beekeeping in the Philippines due to its ease of management and handling [28,32]. However, *A. mellifera* colonies face significant challenges from pests and diseases in the Philippines. Infestations of *Tropilaelaps* mites, *Varroa destructor*, American foulbrood, and small hive beetle (*Aethina tumida*) are common, making beekeeping difficult [28,33]. Despite these pressures, *A. mellifera* outperforms native bees as a pollinator. While *A. cerana* is effective at pollinating native crops, *A. mellifera* is essential for pollinating commercial crops, leading to increased yields and honey production in Asia [34,35].

**Figure 1:**
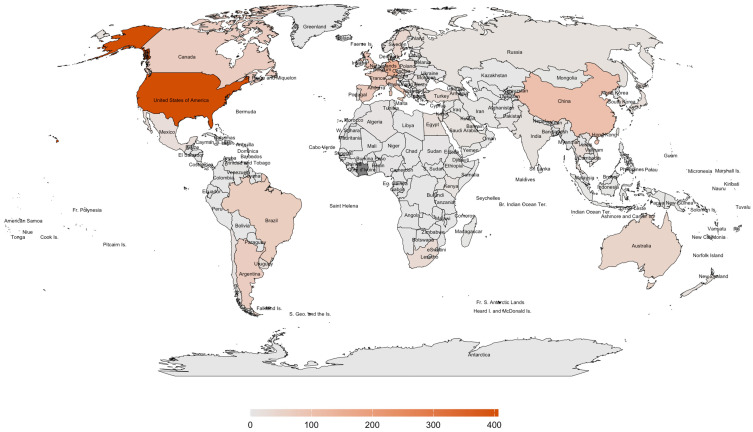
World map showing the global distribution of published articles on the gut microbiome of *Apis mellifera* from 2011 to 2024. Data were extracted from Scopus, and the map was generated using R version 4.3.3 with the packages ggplot2, rnaturalearth, rnaturalearthdata, sf, and dplyr. The number of articles is indicated by color intensity: high (dark orange), low (light orange), and no data (gray).

**Figure 2:**
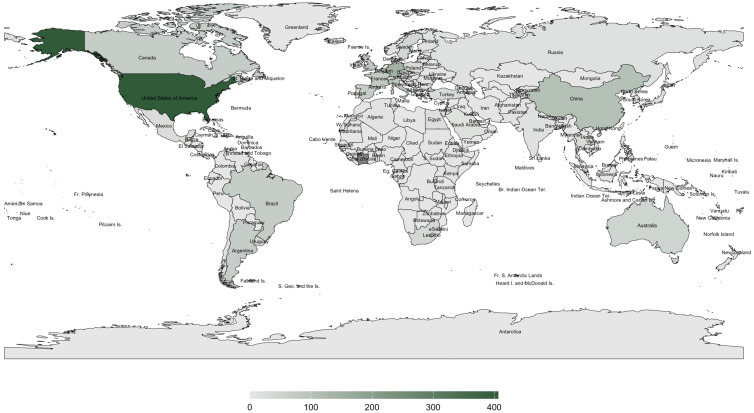
World map showing the global distribution of published articles on pathogens and/or diseases in *Apis mellifera* from 2011 to 2024. Data were extracted from Scopus, and the map was generated using R version 4.3.3 with the packages ggplot2, rnaturalearth, rnaturalearthdata, sf, and dplyr. The number of articles is indicated by color intensity: high (dark green), low (light green), and no data (gray).

This study addresses the critical research gap on *A. mellifera* in the Philippines, particularly regarding the underexplored gut microbiome. The Philippines’ unique geographic features—Luzon, Visayas, and Mindanao, bordered by the Pacific Ocean, South China Sea, and Sulu and Celebes Seas [36]—create distinct microclimates that may influence pollinator health and behavior [37]. These environmental variations are vital in shaping honeybee gut microbiomes, as regional and climatic factors drive microbiome diversity and composition [38,39]. For example, geographic variation has been substantially altering honeybee gut microbial communities, which plays a pivotal role in environmental heterogeneity in fostering microbial diversity [40]. Zhu et al. [41] established a link between geographic variation and microbial diversity in aquatic insects, emphasizing the importance of exploring similar dynamics within the Philippine setting. Given the Philippines’ recognition as a global biodiversity hotspot, understanding the gut microbiome of *A. mellifera* is essential for ensuring pollinator health and supporting conservation and food security efforts [42]. Despite the importance of honeybees in the region, a scoping review of the available literature revealed significant gaps in research on *A. mellifera* in the Philippines. As illustrated in Figure 1 and Figure 2, global studies on the gut microbiome and pathogens/diseases in *A. mellifera* are extensive, but the Philippines remains underrepresented. Existing research focused on stingless bees such as *Tetragonula biroi* [43] or case reports on *Varroa* infestation [44], with limited studies on honeybees, particularly in the *A. mellifera* microbiome. This study represents the first comprehensive investigation into the gut microbiome of *A. mellifera* in the Philippines.

In this study, we hypothesize that there is a significant difference in the microbial communities present in the gut of *A. mellifera* collected from two sites within the same geographical zone in Bacnotan, La Union, Philippines, due to variations in their foraging behavior, diet, and environmental interactions. Understanding the gut microbiome can help identify beneficial microbes that support bee health and productivity. This knowledge can guide beekeepers in developing probiotic supplements to enhance colony health and resilience; given the growing interest in the honeybee gut microbiome and the scarcity of published data on *A. mellifera* in the Philippines, this study aims to elucidate the gut microbiome of *A. mellifera*, focusing on the diversity and abundance of bacterial and fungal communities and their interrelationships.

## 2. Materials and Methods

### 2.1. Honeybee Sample Collection and Site Description

**Figure 3:**
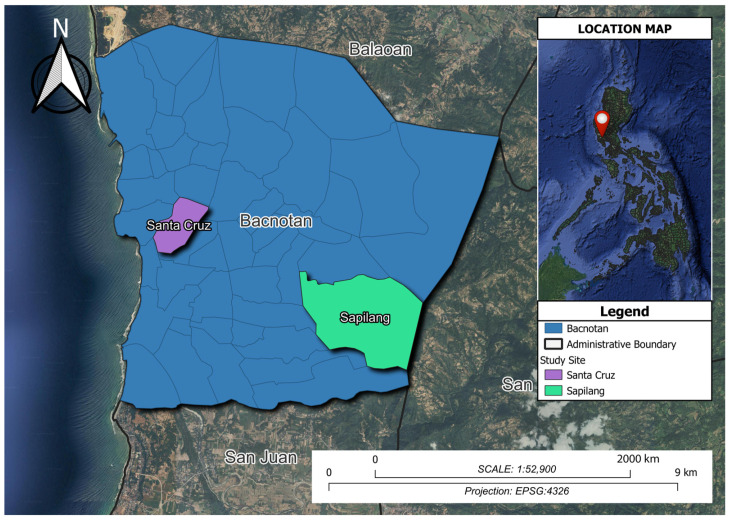
Apiary location in Northern Luzon, located at the Municipality of La Union: Barangay Santa Cruz and Barangay Sapilang.

A total of 400 honeybees were collected in August 2023 from two sites within one geographical location. The first site was the State University apiary in Don Mariano Marcos Memorial State University (DMMMSU), Barangay Sapilang (SP) (16°43’36” N, 120°23’26” E). The second site was a private apiary in Barangay Santa Cruz (SC) (16°44’32” N, 120°20’56” E), Bacnotan, La Union, Philippines (Figure 3). A photograph of the collected honeybee sample is in Supplementary Figure S1. The two sites, while differing in specific landscape features, such as the presence of fruiting trees and proximity to residential areas, share similar ecological resources. Site SC, with fewer fruiting trees and flowers, is adjacent to a residential area with buildings and houses. In contrast, site SP features a more diverse landscape with more fruiting trees, flowers, and vegetation. Despite these differences, both sites offer comparable vegetation and foraging opportunities, with the influence of the nearby coastal environment, the West Philippine Sea, contributing to the availability of ecological resources within their shared radius.

Fifty adult worker honeybees were collected from each of the four colonies per site/apiary. Honeybee samples were placed in sterile 50 mL conical tubes. Subsequently, *RNAlater* (Thermo Scientific Inc., Waltham, MA, USA) solution was added to the tubes to preserve the RNA and DNA of the samples. The honeybee samples were transported under chilled conditions using an ice box to the laboratory at Chiang Mai University, Chiang Mai, Thailand, and stored at −80 °C for further analysis.

### 2.2. Sample Preparation and Genomic DNA Extraction

Honeybee samples were surface-sterilized following the method of Pakwan et al. [45] with some modifications. The honeybee samples were immersed in 7% (*v*/*v*) sodium hypochlorite for 1 min and 70% (*v*/*v*) ethanol for 3 min, then soaked and rinsed three times in sterile distilled water and dried on sterile paper towels. Using sterilized forceps and a scalpel, the head and thorax were separated from the abdomen. An incision was made in the abdomen to collect the honeybee’s gut. The dissected guts were pooled [11] and then placed in a ZR BashingBead™ lysis tube containing ZymoBIOMICS™ lysis solution (ZYMO Research, Freiburg im Breisgau, Germany), then lysed for 20 min using a Disruptor Genie (Scientific Industries, Bohemia, NY, USA).

For genomic DNA extraction, we prepared pooled samples from eight separate colonies, with each sample consisting of ten adult workers of honeybees per colony. Four colonies were sampled from site SP, and four are from site SC to ensure representation of both locations. We pooled the dissected guts to capture more comprehensive representation of the microbiota within the colony and mitigate individual variations. Adult worker honeybees of similar age were selected to control age-related differences in microbiome composition. Total genomic DNA was extracted from each of these samples using the manufacturer’s protocol and was provided with the ZymoBIOMICS™ DNA Miniprep Kit (ZYMO Research, Germany). The DNA concentration was determined using a NanoDrop UV–vis spectrophotometer (Thermo Fisher Scientific, Madison, WI, USA). 

### 2.3. Bacterial 16S rRNA Gene Amplicon and Fungal ITS Gene Amplicon Sequencing

The extracted DNA was sent to Macrogen, Inc. (Seoul, Republic of Korea), for library construction and sequencing on an Illumina paired-end MiSeq platform. The 16S rRNA fragment was amplified using specific primers: 341F (5′-CCTACGGGNGGCWGCAG-3′) and 805R (5′-GACTACHVGGGTATCTAATCC-3′) [46]. For the ITS region, amplification was conducted with the forward primer ITS1F (5’-CTTGGTCATTTAGAGGAAGTAA-3’) [47] and the reverse primer ITS2 (5’-GCTGCGTTCTTCATCGATGC-3’) [48]. The raw NGS reads (FASTQ files) were subjected for analysis. These reads were then processed using QIIME2 version 2024.10 [49]. Primer sequences were trimmed, and reads were quality-filtered, truncating at positions where the Phred score was below 30. Quality filtering and denoising were performed using DADA2 [50]. Singletons were subsequently removed [51]. Rarefaction curves were generated to determine the appropriate depth, and datasets were standardized to a different depth of sequences per sample [52]. Taxonomic classification was performed using the SILVA 138 database [53] and the UNITE database version 8.3 [54], with a naive Bayes classifier assigning taxonomy to the 16S rRNA and ITS sequences, respectively.

### 2.4. Bioinformatic and Statistical Analyses

The diversity of the bacterial and fungal microbiome was analyzed using the ’vegan’ package in R version 4.3.3. To measure alpha diversity, the Shannon [55] and Simpson [56] indices were employed as they provide insights into the evenness and richness of the microbiome [57].

The processed NGS data was analyzed using the Mann–Whitney U Test to compare the alpha diversity of *A. mellifera* species across two locations. Bacterial and fungal communities were analyzed using Bray–Curtis dissimilarity metrics [58]. Group differences were evaluated using an analysis of similarities (ANOSIM) and visualized through non-metric multidimensional scaling (NMDS) and principal coordinate analysis (PCoA) through R version 4.3.3.

Functional pathways were predicted using the ENZYME nomenclature database with PICRUSt2 software version 2.5.2 [59]. A heatmap was generated to display the hierarchical clustering of each predicted gene, utilizing R via RStudio [60,61]. Correlations in bacterial and fungal taxonomy were examined using Spearman’s correlation through the ’Hmisc’ package in R. Only significant correlations (*p* < 0.05) with strong coefficients were imported into Gephi 0.9.2 [62] for visualization using the Fruchterman–Reingold layout.

## 3. Results

### 3.1. Sequence Read Curation

Targeted amplicon sequencing for 16S rRNA resulted in 39,109 minimum paired-end reads in a demultiplexed sequence count summary. After quality control filtering, denoising, and removal of chimeric sequences, we obtained 184,633 amplicons. Following this, after removing singletons, a total of 149,528 amplicons were generated with a minimum of 18,691, corresponding to 374 unique ASVs. Conversely, the ITS amplicon dataset resulted in 41,252 minimum paired-end reads in a demultiplexed sequence count summary. After quality control and removal of chimeric sequences, a maximum and minimum of 40,985 and 29,842 amplicons, respectively, were generated. Rarefaction analyses were conducted, and adequate sample sizes, indicated by plateau curves, accounted for 238,736 unique amplicons corresponding to 475 unique ASVs. All demultiplexed count summaries and rarefactions are listed in Supplementary Tables S1 and S2.

The 16S rRNA and ITS sequencing showed the types and diversity of microbes in the sampled environment. After careful processing, a robust set of high-quality bacterial sequences was identified, highlighting the well-defined diversity in the sample [63,64,65].

The rarefaction curves reached a plateau (Supplementary Figure S2), indicating that the sampling sizes, referring to the number of sequences obtained from NGS, have captured the major diversity of the microbial communities, and additional sequencing is unlikely to reveal significantly more diversity.

### 3.2. The Gut Bacteriota of Apis mellifera

**Figure 4:**
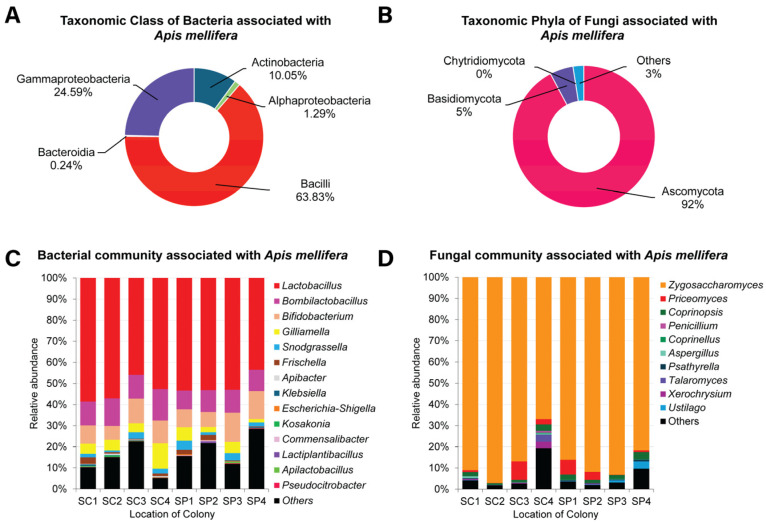
Taxonomic class and phyla associated in *Apis mellifera* (**A**,**B**). The gut bacteriome (**C**) and gut mycobiome (**D**) of *Apis mellifera* from the two locations from the Municipality of La Union, Philippines: Barangay Santa Cruz (SC1–SC4) and Barangay Sapilang (SP1–SP4). Percentage of relative abundance was shown. Features were clustered and organized (colored taxa bar plots) based on genus. Abundance with <0.1% is clustered into “Others”.

Variations in bacterial community associated with *A. mellifera* were observed among all colonies. Based on the NGS results, bacteriomes are composed of 25 genera belonging to 4 phyla, 5 classes, 9 orders, and 16 families. The bacterial community under taxonomic class (Figure 4A) in both sites (SC and SP) were dominated by the class Bacilli, accounting for 63.81%, followed by γ-proteobacteria (24.58%), Actinobacteria (10.05%), α-proteobacteria (1.29%), and Bacteroidia with <1% relative abundance. Figure 4B presents the percentage of relative abundance of each bacterial genera associated with *A. mellifera*. Within the phylum of Firmicutes and class Bacilli, the most abundant representative genus was *Lactobacillus*, accounting for 52.13% of the community, followed by *Bombilactobacillus* (11.34%), while the species *Apilactobacillus* and *Lactiplantibacillus* constituted less than 1% of the percentage of relative abundance. Within γ-proteobacteria, the most representative genus was *Gilliamella* with 5.24% abundance, followed by *Snodgrassella* (2.34%) and *Frischella* (1.31%). Other genera with <1% relative abundance were *Escherichia–Shigella*, *Klebsiella*, *Kosakonia*, and *Pseudocitrobacter*. Meanwhile, Actinobacteria comprised the most abundant genera, *Bifidobacterium* and *Corynebacterium*, with bacterial relative abundances of 10.044% and 0.002%, respectively. In contrast, α-Proteobacteria was represented solely by the genus *Commensalibacter* (0.13%). Lastly, the class Bacteroidia included only the genus *Apibacter*, with a relative abundance of 0.24% across the dataset. Comparing the bacterial communities at the two sampling sites, SC and SP, reveals no appreciable differences in composition.

### 3.3. The Gut Mycobiota of Apis mellifera

Overall, the metagenomic results revealed the presence of 100 fungal genera belonging to 3 phyla, 13 classes, 27 orders, and 73 families. The most representative phylum was Ascomycota, accounting for 92.24% of all datasets, followed by Basidiomycota (5.32%) and Chytridiomycota (less than 1%) (Figure 4C). Within the taxonomic classes, Saccharomycetes was the most abundant, representing 90.42%, followed by Agaricomycetes (3.02%), Ustilaginomycetes (1.94%), and Eurotiomycetes (1.52%). Other classes were present with less than 1% abundance. Among the Saccharomycetes, the most representative genus was *Zygosaccharomyces* with 86.81% relative abundance (Figure 4D). Additionally, *Priceomyces* had a relative abundance of 2.96%. Other genera such as *Coprinopsis*, *Ustilago*, *Xerochrysium*, *Talaromyces*, *Psathyrella*, *Aspergillus*, *Coprinellus*, and *Penicillium* were also detected. Similar to bacterial community, each location does not differ significantly based on fungal composition.

### 3.4. Differences in Diversity Across Site Locations

**Figure 5:**
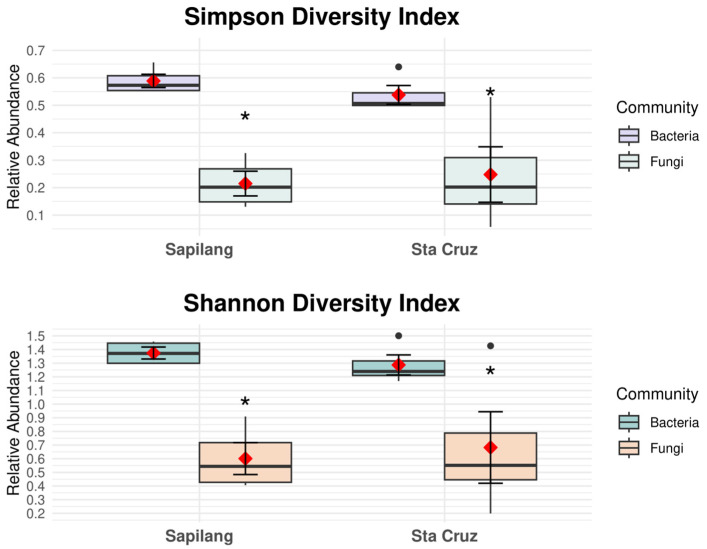
Alpha diversity plots. Boxplot corresponding to Shannon diversity and Simpson diversity index. Taxonomic, bacterial community, and fungal community have no significant evidence (*p* < 0.05, Mann–Whitney U Test). Mean data correspond to the red polygon point, and the standard error is represented as the asterisk, and black dots represent individual data points.

Figure 5 depicts the Shannon (A) and Simpson (B) diversity of the bacterial community of *Apis mellifera* from two site locations in La Union, Philippines. At the cut-off significance level of 0.05, the results showed no significant differences in α diversity for Shannon diversity (*p* = 0.20) and Simpson diversity (*p* = 0.34), as measured by Mann–Whitney U tests. Likewise, the α diversity for the fungal community and Shannon and Simpson diversities have a *p*-value of 1.00. Therefore, there was no difference in the diversity of the fungal community associated with *A. mellifera* between the two sites.

The analysis of β diversity, both overall and pairwise, using the ANOSIM test revealed no significant differences in the composition of bacterial or fungal communities between the two sampled locations. Specifically, the R values for both bacterial (R = 0.01) and fungal (R = −0.07) communities were close to a zero value, indicating minimal differentiation between the two groups. On the other hand, *p*-values for bacteria (*p* = 0.45) and fungi (*p* = 0.74) were both greater than the significance threshold of 0.05. This finding suggests that the gut microbiomes of *A. mellifera* are relatively conserved across different environmental settings. The ANOSIM result indicates that there is little to no differentiation between the sites.

The results are visualized using non-metric multidimensional scaling (NMDS) for bacterial (Supplementary Figure S3A) and fungal (Supplementary Figure S3B) communities. The stress values of bacterial and fungal communities were less than 0.2 (9.4 × 10^−5^ and 8.2 × 10^−5^, respectively).

The results show that the bacterial (Supplementary Figure S4A) and fungal ASVs (Supplementary Figure S4B) in the two locations are similar, with overlapping points, ellipses, and hulls indicating no significant differences in microbial communities. Potential sources contributing to this similarity were also identified.

### 3.5. Functional Properties of the Gut Microbiome of Honeybee, Apis mellifera

**Figure 6:**
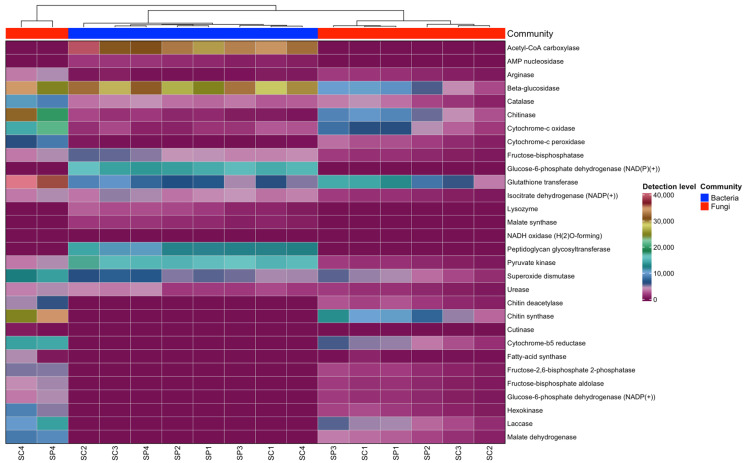
Heatmap visualization for significantly differential functional metabolites enzymes identified in the gut microbiome of *Apis mellifera*. Colors indicate the detection level of functional metabolite enzymes, ranging from highest (light pink, red) to lowest (dark purple): pink-red indicates a relatively higher level, dark purple indicates a relatively lower level, and light green indicates that the relative level of metabolites falls between high and low.

The predicted key enzymes in the gut microbiome of *A. mellifera* were β-glucosidase, chitinase, glucose-6-phosphate dehydrogenase, cytochrome enzymes, glutathione transferase, AMP nucleosidase, lysozyme, malate synthase, laccase, hexokinase, and superoxide dismutase, identified in both bacterial and fungal microbiomes (Figure 6).

### 3.6. Correlations of Bacterial and Fungal Microbiome

**Figure 7:**
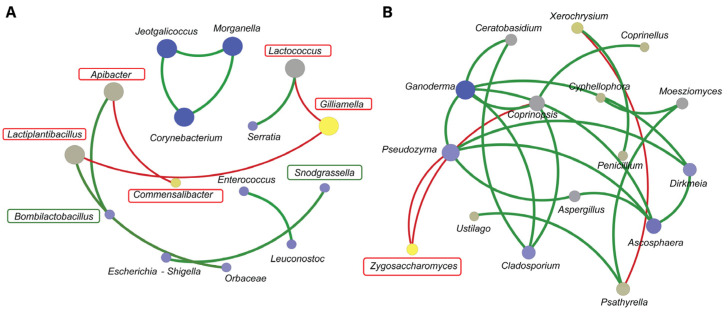
The network analysis of bacterial and fungal communities reveals several correlations. For the bacterial community (**A**), there is a negative correlation between *Commensalibacter* and *Apibacter*, *Gilliamella* and *Lactococcus*, and *Gilliamella and Lactiplantibacillus.* A positive correlation was observed between *Snodgrassella* and *Escherichia–Shigella* and *Bombilactobacillus* and *Apibacter.* For the fungal community (**B**), *Zygosaccharomyces* shows a negative correlation with *Coprinopsis* and *Pseudozyma.* The degree of correlation was evaluated using Spearman’s correlation (*p* < 0.05) and represented by the color of the edges, ranging from positive (red) to negative (green), and by the size of the nodes, indicating the strength of the correlation from strong (yellow) to weak (blue).

There was a correlation between gut microbiomes of bacteria and fungi and their potential environmental influences. The correlation plot revealed 15 nodes and 11 edges within bacterial communities (Figure 7A), highlighting specific interactions: *Commensalibacter* was negatively correlated with *Apibacter*, while *Gilliamella* showed negative correlations with *Lactococcus* and *Lactiplantibacillus*. In contrast, *Bombilactobacillus* exhibited a strong positive correlation with *Apibacter*, and *Snodgrassella* showed a strong positive correlation with *Escherichia–Shigella*. The size of the nodes indicated the degree of interaction, mainly suggesting positive interactions within the community.

Conversely, the fungal community (Figure 7B) exhibited 16 nodes and 21 edges, with *Zygosaccharomyces* showing negative correlations with *Coprinopsis* and *Pseudozyma*. Positive correlations were more prevalent among fungi, such as *Penicillium’s* positive correlation with *Xerochrysium*, which was negatively correlated with *Psathyrella*. Notably, *Ganoderma* was positively correlated with *Dirkmeia*, *Ceratobasidium*, *Coprinopsis*, and *Ascosphaera*, with strong interaction levels observed. These findings suggest that environmental factors like food resources and habitat influence gut community composition and alter the relationships within these communities.

## 4. Discussion

Analyzing the gut microbiome of *Apis mellifera* using next-generation sequencing (NGS) is crucial for understanding microbial diversity and its impact on bee health and management. By examining functional pathways and enzymes, this study sheds light on how different microbes influence bee metabolism, digestion, and immunity, guiding strategies to enhance bee health and productivity. Additionally, employing Spearman correlation to explore relationships among various taxa helps identify key microbial interactions within the gut.

### 4.1. Environmental Factors Shape Microbial Community Similarities Between Sites

Our findings indicate that the most abundant bacterial and fungal communities in *A. mellifera* were similar between the two locations, likely due to the shared ecological resources within the 3–5 km sampling radius [66]. Despite differences in landscape features—such as fruiting trees and proximity to residential areas—both sites offer comparable vegetation and foraging opportunities. The nearby coastal environment, particularly the West Philippine Sea, further contributes to the availability of ecological resources in both Barangay Sapilang (SP) and Barangay Santa Cruz (SC). As a result, the honeybees from both sites exhibited a similar microbiome composition, with no significant differences in the Shannon and Simpson diversity indices. These findings are consistent with previous studies, such as Cohen et al. [67], which demonstrated that environmental factors like floral availability and landscape structure heavily influence the bee microbiome. The similarities in the gut microbial communities at both sites suggest that these apiary areas’ environmental conditions and foraging resources are sufficiently alike for supporting comparable bacterial and fungal populations in the honeybees.

### 4.2. Characterization of the Core Gut Microbiome and Fungal Diversity in Apis mellifera

Our findings confirm that the gut microbiome of *A. mellifera* includes a core microbiome characterized by a high abundance of Gram-positive species. As shown in the taxonomy stacked bar charts (Figure 4C,D), the most prevalent bacterial community belonged to the phylum Firmicutes, particularly the class Bacilli, with *Lactobacillus* accounting for 52.13% of the bacterial population. Additionally, we found a significant presence of *Bombilactobacillus* and *Bifidobacterium* across both locations. *Lactobacillus*, *Bombilactobacillus*, and *Bifidobacterium* are vital members of the bee gut microbiome, thriving in the honeybee digestive system [5,9,68]. Interestingly, our data included the genus *Bombilactobacillus.* This genus, recently reclassified, was previously grouped with *Lactobacillus* species associated with social bees and has been identified in the stomach and hindgut of honey bees (*A. mellifera*), but was later established as a distinct genus under *Bombilactobacillus* [69]. Additionally, *Apilactobacillus*, although present at lower abundances, was also detected in our samples. Notably, *Lactobacillus kunkeei*, commonly found in honey, bee products, and bee-related crops, has been reassigned from its original classification under *Lactobacillus* to the genus *Apilactobacillus*. It is now recognized as a heterofermentative sister genus of *Bombilactobacillus* [69,70]. Notably, our results are consistent with those of Ellegaard et al. [71], who also reported a core microbiome in both *A. mellifera* and *A. cerana*. This reinforces our findings that *A. mellifera* from the Philippines maintains a conserved core gut microbiome, providing a valuable foundation for further research on the gut microbiome of honeybees in the Philippines.

We also identified members of γ-proteobacteria, such as *Snodgrassella*, *Gilliamella*, and *Frischella*, and α-proteobacteria, including *Commensalibacter*. Although these species were present in lower abundances across both locations, growing evidence suggests that even these less dominant members play a crucial role in the gut microbiome of *A. mellifera*. Similar findings regarding the core gut microbiome of *A. mellifera* have been reported [39,71,72,73].

The diversity of fungi in the gut of *A. mellifera* did not vary significantly between the two locations. Both locations showed a similar proportion of fungal communities dominated by yeasts from the genus *Zygosaccharomyces* under the phylum Ascomycota. The common fungi associated with bees belong to the phylum Ascomycota [18,27]. Similar results were observed in the gut communities of *A. mellifera* in South Korea, which were also colonized by abundant *Zygosaccharomyces* [74]. Rutkowski et al. [27] also reported that the common genera of yeasts associated with bees include *Starmerella*, *Metschnikowia*, *Zygosaccharomyces*, and *Candida*. Conversely, some studies suggest that the association of yeasts and bees may originate from other sources. For instance, *Zygosaccharomyces* has been consistently found in abundance in the honey of *A. mellifera* [24,75]. This implies a mutualistic interaction between yeasts and honeybees [26,76]; as such, increased yeast levels in bee nourishment indicate the metabolic activity of these micro-organisms [77].

We also found low abundances of *Priceomyces*, *Coprinopsis*, *Ustilago*, *Xerochrysium*, *Talaromyces*, *Psathyrella*, *Aspergillus*, *Coprinellus*, *Penicillium*, and other fungi distributed across both locations. Bees acquire these fungi from floral resources [27] and sometimes from hive-stored bee bread [78] or the hive environment, as with *Penicillium* species [79]. It has been reported that *Aspergillus* spp. can be opportunistic and compromise the immune system, although the severity of its effects varies significantly [80]. While we did not investigate the mode of transmission or the effects of these fungi on the immune system of honeybees, it is noteworthy that despite their potential negative impacts, these fungi might be parasitic, commensal, or mutualistic [80]. This highlights the notion that diverse fungi, like bacterial communities, colonize the gut of honeybees.

### 4.3. Ecological and Geographical Factors Contributing to Gut Microbiome Similarity

Our diversity measures are relative, and although we initially expected differences between the two locations, the results indicate that the microbiomes of *A. mellifera* from both sites are remarkably similar. This similarity is supported by non-metric multidimensional scaling and principal coordinate analysis. While the sites exhibit distinct landscape features, site SC is adjacent to residential buildings with fewer fruit trees, while site SP has more diverse vegetation; both locations benefit from the nearby coastal environment, particularly the West Philippine Sea. This likely contributes to similar abiotic conditions across the two sites despite the lack of direct measurements of humidity and temperature in this study. We hypothesize that the coastal influence plays a significant role in maintaining comparable core gut microbiota in the honeybees, mitigating the potential effects of the landscape differences.

A study from Sussex, UK, found no significant difference in the gut microbiome diversity of honeybees from two different landscape types: farmland with oilseed rape flowers and agricultural land distant from oilseed rape flowers [81]. This suggests that similar microbiota can exist across various sites. Additionally, because we are studying a single species of honeybee, *A. mellifera*, the gut microbiome might be similar regardless of location. As demonstrated by Martinson et al. [82], *A. mellifera* is consistently colonized by a distinctive set of bacterial species across different locations. Although their study focused on bacteria, a similar phenomenon may occur within the fungal community. Moreover, the gut communities of *A. mellifera* show significant ecological resilience, maintaining a unique group of organisms across varied environmental conditions, both among and within individual bees [8]. Factors such as food resources and natural flora should also be considered.

Additionally, the proximity of the sites, within a 3–5 km radius, aligns with the typical foraging range of honeybees, which can exceed 5 km [83]. Therefore, it is unsurprising that the proportions of bacterial and fungal communities are similar between the two sites. Nevertheless, our study provides valuable insights into the gut microbiome of *A. mellifera* in the Philippines and offers the first general overview of this microbiome in the region.

### 4.4. Predicted Functional Enzymes and Their Roles in Honeybee Health

Our metagenomic research also identified functional gene contents related to key enzymes, which provided new insights into taxonomic diversity and microbial community structures beyond traditional 16S rRNA and ITS analyses. Our findings highlight several significant enzymes in bacterial and fungal communities that play crucial roles in host defense, carbohydrate metabolism, and energy regulation. For example, antimicrobial peptides (AMPs) detected within the bacterial community are vital for honeybee immune defense against pathogen invasion [84]. Furthermore, key enzymes such as β-glucosidase, catalase, glutathione transferase, and superoxide dismutase (SOD) were observed in both microbial communities. These enzymes contribute to the defense system and help maintain metabolic processes in the host. For instance, in the insect *Aphis pomi*, increased enzymatic activity was reported in response to varying temperatures [85]. Although this was specifically observed in *A. pomi*, we can infer a similar response in honeybees, suggesting that these insects also activate these enzymes as part of their innate immune response to thermal stress.

Interestingly, while enzyme activity in *A. pomi* showed notable changes with temperature variations, studies on the Egyptian honeybee (*A. mellifera lamarckii*) and the European honeybee (*A. mellifera carnica*) revealed different metabolic responses. *A. mellifera lamarckii* exhibited minimal variations in heat emission, whereas *A. mellifera carnica* showed a significant reduction in metabolic rate under elevated temperatures [86]. Lysozymes and glucose-6-phosphate dehydrogenases are key carbohydrate-metabolizing enzymes identified in *A. mellifera* [87]. Although our study did not directly examine host interactions with enzyme function predictions, it is noteworthy that the gut microbiota harbors these essential enzymes. Our bioinformatics analysis provides valuable insights into the predicted functional enzymes present in the gut community, which are crucial for carbohydrate metabolism, energy absorption, and host defense. However, because these enzymes were predicted using PICRUSt2 based on 16S rRNA and ITS data, which provide significant information into microbial composition, further research and validation are needed to explore the functional roles through metagenomic and metatranscriptomic techniques. Such investigations would enable the elucidation of metabolic or transcriptional changes related to the host’s innate immune system response.

### 4.5. Microbial Association Network Support the Bee’s Immune Defense

Various bee symbionts within our samples exhibited negative correlations with other bacterial taxa. For instance, *Commensalibacter* showed a negative correlation with *Apibacter* while *Gilliamella* were negatively correlated with *Lactococcus* and *Lactiplanbacillus*, respectively. In addition, *Snodgrassella* positively correlated with *Escherichia–Shigella*, while *Bombilactobacillus* showed a positive correlation with *Apibacter*. Previous studies have indicated that *Gilliamella* exhibits negative associations with other micro-organisms, suggesting that host immune-induced disruptions may affect them [88]. Although studies about *Bombilactobacillus* is limited, the positive associations from our analysis might suggest a fermentation process role within the gut. As a member of the lactic acid bacteria group, it could contribute to the production of secondary metabolites, which are critical for microbiome and host health [89,90]. Furthermore, bees with a complete gut microbiome, such as those containing *S. alvi* or *G. apicola*, were more effective at eliminating bacteria from the hemolymph following *E. coli* exposure and exhibited higher levels of antimicrobial peptides (AMPs) compared with bees lacking a microbiome [6]. This suggests that these core gut microbiota members contribute to immune priming.

As for fungi, *Zygosaccharomyces* was negatively correlated with *Coprinopsis* and *Pseudozyma*. The abundance of *Zygosaccharomyces* increased markedly when exposed to a high dose of fluvalinate stress [91]. This indicates that while the metabolism of honeybees is affected, the immune response continues to protect the host against induced stress. Although these mechanisms were not the focus of our experiment, we highlight the relationship between essential bee symbionts and other microbiome members. Further research is needed to elucidate the specific mechanisms of synergistic resistance between the bee body and gut microbiota in response to different stressors.

### 4.6. Building Baseline Data for Honeybee Microbiome in a Unique Environment in the Philippines

This study underscores the baseline data of microbiome research, particularly in a region like the Philippines, where such studies are limited. The European honeybee, *A. mellifera*, was introduced to the Philippines by C.H. Schultz in 1913 [92,93,94]. Beekeeping management only gained popularity in the 1990s, in which colony numbers have remained relatively low [95]. The Food and Agriculture Organization of the United Nations reported in 2021 that only an average of 40 colonies were documented in the Philippines [96]. These figures highlight the importance of baseline data into the exploration of microbiome studies in this region. Microbial communities in honeybees play a pivotal role in maintaining bee health, influencing digestion and overall resilience. This context is important in terms of environmental challenges. For instance, a high heat index was experienced in the Philippines in 2023. Our sampling in August 2023 coincided with elevated ambient temperatures and low relative humidity in Ilocos Norte (Northern Luzon, Philippines) [97]. Such environmental conditions can directly influence the diversity of the gut microbiome, shaping its ability to adapt to extrinsic factors.

Similarly, studies have shown that the composition and diversity of the gut microbiome in bees are closely linked to the quality and variety of their diet [98,99]. A diverse, nutrient-rich diet from various floral resources supports a healthy and balanced gut microbiome. The gut microbiota also adapts to dietary changes, highlighting the connection between bee nutrition and gut health [98,99]. Given the limited number of colonies in the region, combined with the unique environment of the Philippines, establishing studies like this is essential for promoting effective beekeeping management practices. In addition, Figure 2 highlights the global distribution of published data on pathogens and diseases in *A. mellifera*, revealing significant research gaps in regions like the Philippines. Future studies should address these gaps by expanding sampling efforts that include native bee species and conducting exploratory evaluations of pathogens, such as microsporidian parasites, viruses, and others. These efforts would contribute to a broader understanding between microbiomes, environmental conditions, and beekeeping practices in the Philippines.

## 5. Conclusions

Our study comprehensively characterizes the microbial communities in association with *Apis mellifera* honeybee from the Philippines. Although, the gut microbiomes showed similar proportions across two locations, suggesting that the microbiome is shaped by location, diet, and food resources. In addition to gut microbiome data, we identified predicted functional enzymes linked to host defense and metabolism, highlighting their critical role in supporting honeybee health. These findings underscore the importance of understanding microbiome dynamics in honeybee health and resilience, with future research needed to explore temporal variations and the impact of environmental or hive management factors on pathogen presence and microbiome functionality.

## Data Availability

The raw sequencing data presented in this study were uploaded to the National Center for Biotechnology Information (NCBI) under BioProject PRJNA1140353.

## Supplementary Materials

The following supporting information can be downloaded at: https://www.mdpi.com/article/10.3390/insects16020112/s1, Figure S1: Collection of samples in honeybee cages. Figure S2: Rarefaction curves of the bacterial and fungal community generated via QIIME2. Figure S3. Distances among bacterial and fungal communities in the gut microbiome represented as NMDS plots of Bray–Curtis weighted distances. Figure S4: Principal coordinate analysis of bacterial and fungal community. Table S1: Demultiplexed sequence counts of bacteria generated via QIIME2. Table S2: Demultiplexed sequence counts of fungi generated via QIIME2.
