# Supplementary material for: Gut Microbiome Diversity in European Honeybees (Apis mellifera L.) from La Union, Northern Luzon, Philippines
Source: Insects. 2025 Jan 23;16(2):112. doi: 10.3390/insects16020112 (PMC11855267; doi:10.3390/insects16020112)
Supplement: Supplementary file 1 [file insects-16-00112-s001.zip › insects-3369509-supplementary.pdf]

Supplementary Material, Article

Gut Microbiome Diversity in European Honeybees (*Apis mellifera* L.) from La Union, Northern Luzon, Philippines

Diana Castillo [1,2], Evaristo Abella [2], Chainarong Sinpoo [1,3,4], Patcharin Phokasem [1,3,4], Thunyarat Chantaphanwattana [1,3], Rujipas Yongsawas [1,3,4], Cleofas Cervancia [5], Jessica Baroga-Barbecho [5], Korrawat Attasopa [3,6,7], Nuttapol Noirungsee [1,3,7,*] and Terd Disayathanoowat [1,3,7,*]

[1] Department of Biology, Faculty of Science, Chiang Mai University, Chiang Mai 50200, Thailand; dccastillo@clsu.edu.ph (D.C.); chainarong_s@cmu.ac.th (C.S.); patcharin.ph@cmu.ac.th (P.P.); thunyarat.chan@gmail.com (T.C.); r.yongsawas@gmail.com (R.Y.)

[2] Department of Biological Sciences, College of Science, Central Luzon State University, Science City of Muñoz, Nueva Ecija 3120, Philippines; eaabella@clsu.edu.ph

[3] Research Center of Deep Technology in Beekeeping and Bee Products for Sustainable Development Goals (SMART BEE SDGs), Chiang Mai University, Chiang Mai 50200, Thailand; korrawat.a@cmu.ac.th

[4] Office of Research Administration, Chiang Mai University, Chiang Mai 50200, Thailand

[5] Bee Program, University of the Philippines–Los Baños, Laguna 4031, Philippines; cleofascervancia@gmail.com (C.C.); jbbaroga@up.edu.ph (J.B.-B.)

[6] Department of Entomology and Plant Pathology, Faculty of Agriculture, Chiang Mai University, Chiang Mai 50200, Thailand

[7] Center of Excellence in Microbial Diversity and Sustainable Utilization, Faculty of Science, Chiang Mai University, Chiang Mai 50200, Thailand

* Correspondence: nuttapol.n@cmu.ac.th (N.N.); terd.dis@cmu.ac.th (T.D.)

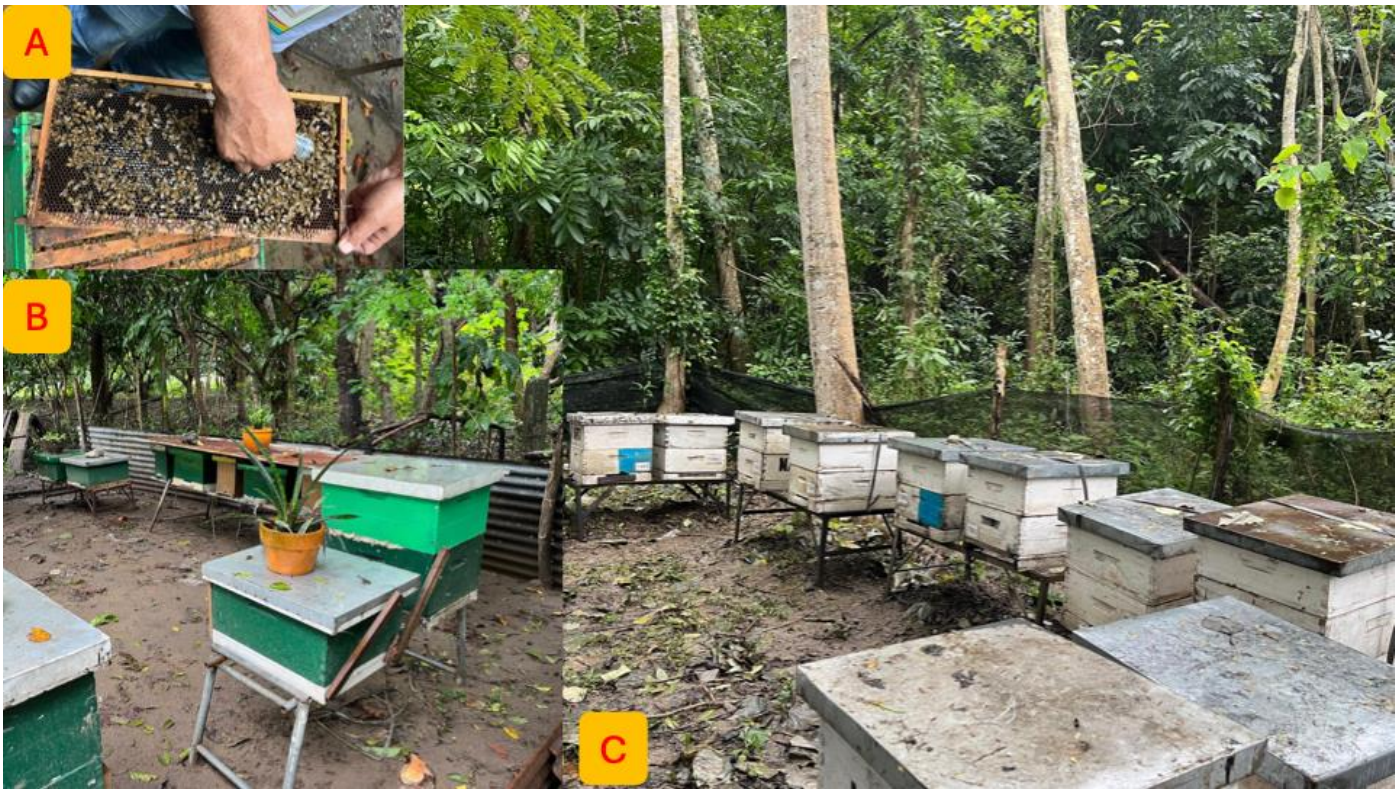

Supplementary Figure S1. Collection of *Apis mellifera* adult bees using a 50-mL conical tube **(A)**, showing honeybee cages from Bacnotan, La Union: Barangay Santa Cruz **(B)** and Barangay Sapilang **(C).** The photograph displays the collection process, and the cages used at these locations.

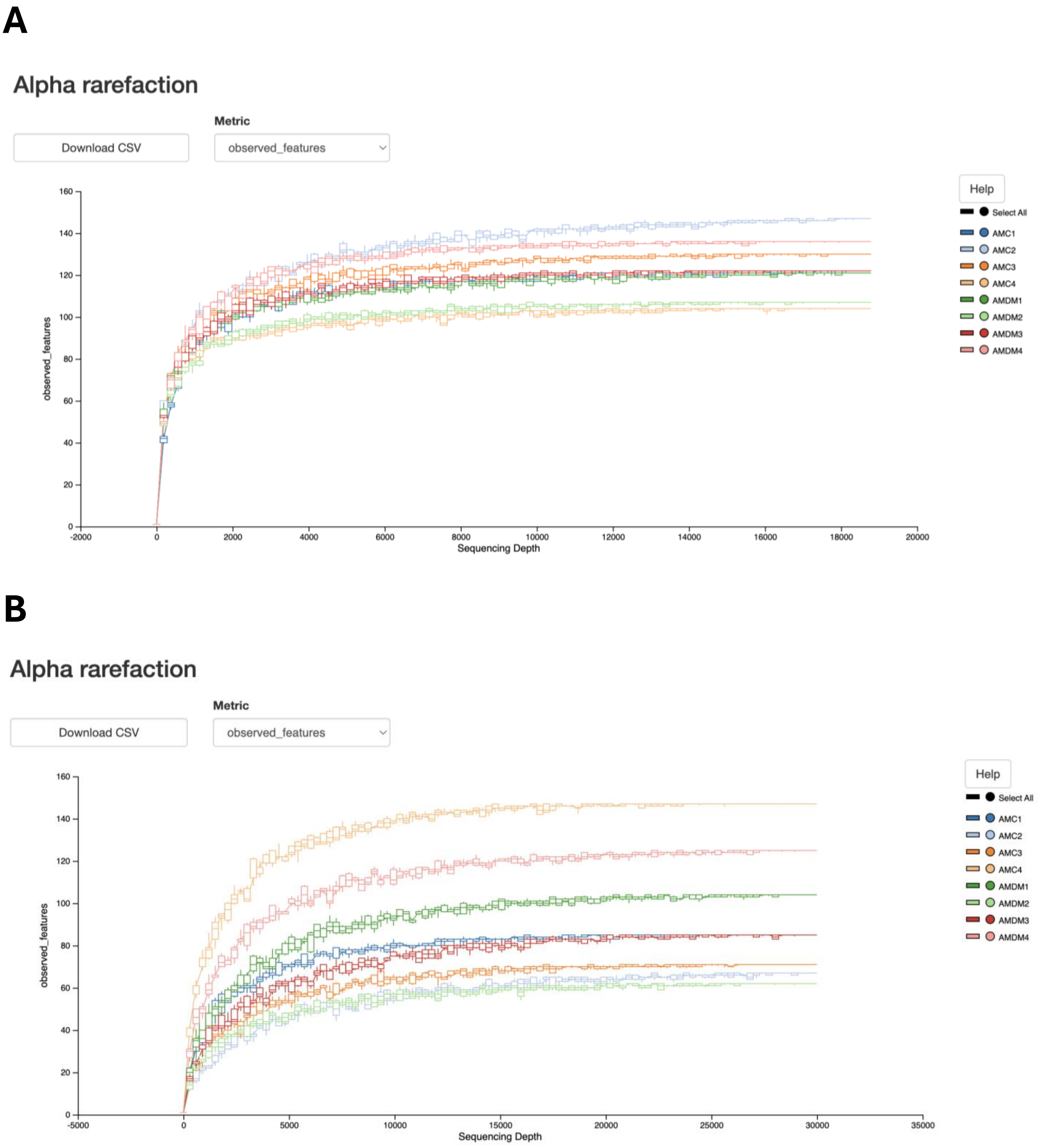

Supplementary Figure S2. Rarefaction curves of bacterial **(A)** and fungal **(B)** communities generated from processed QIIME2 data, showing that sequencing depth reached a plateau. This indicates that the data is sufficient for further analysis.

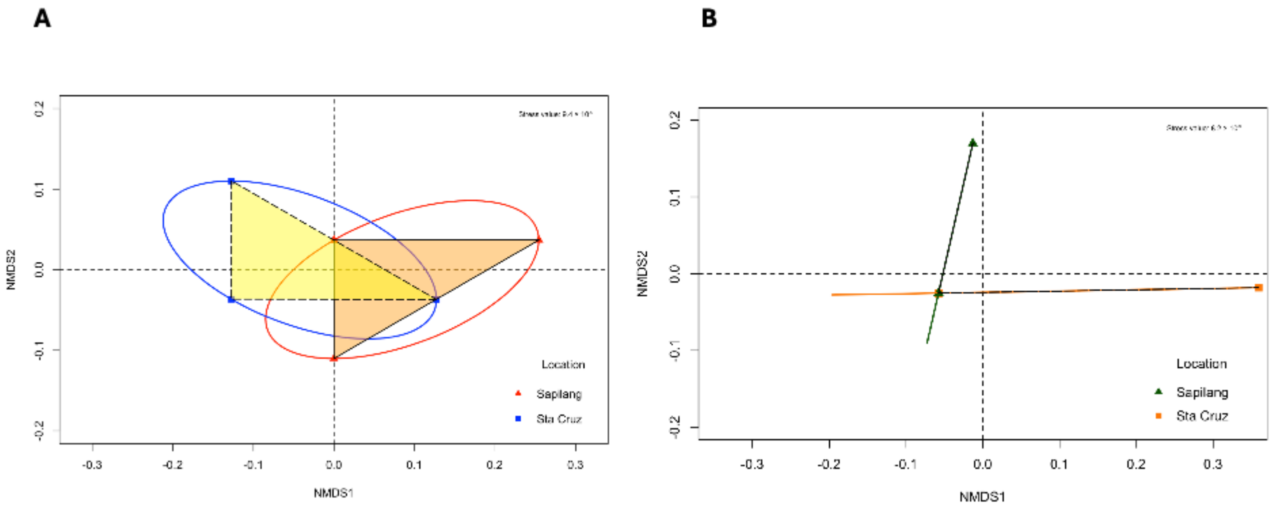

Supplementary Figure S3. Distances among bacterial and fungal communities in the gut microbiome of *Apis mellifera*, represented as NMDS plots of Bray – Curtis weighted distances. NMDS of gut bacterial community **(A)** presented on the blue ellipse shows the area of plot located in Barangay Santa Cruz. The red ellipse shows the area dominated by Barangay Sapilang. **(B)** NMDS of the gut fungal community from Barangay Santa Cruz (orange ellipse) and Barangay Sapilang (green ellipse).

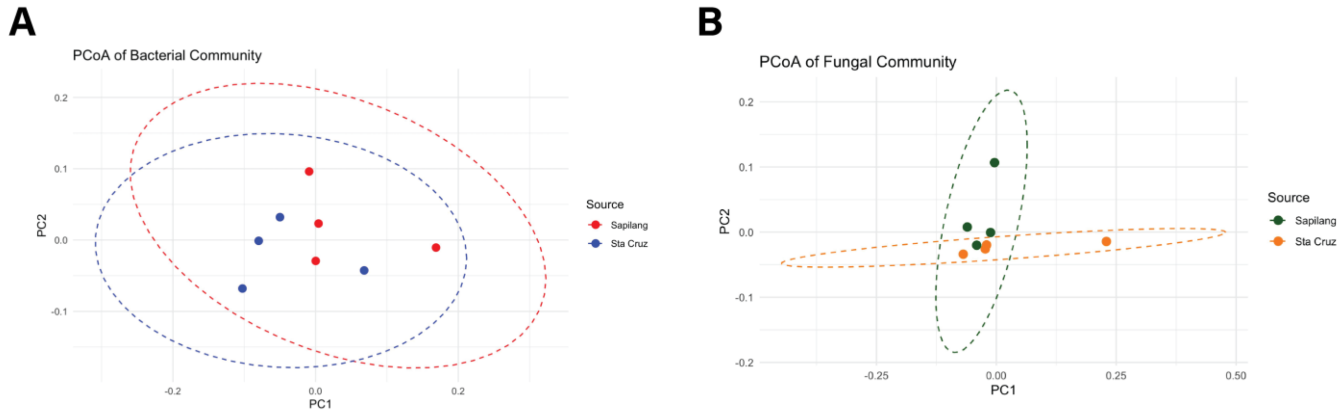

Supplementary Figure S4. Principal coordinate analysis of bacterial **(A)** and fungal **(B)** communities . Ellipses represent 95% confidence ellipses on the ordination. Sapilang: Barangay Sapilang; Sta Cruz: Barangay Santa Cruz.

Supplementary Table S1. Demultiplexed sequence counts and table summary of each frequency dataset and rarefaction from QIIME2 of Bacteria associated with *Apis mellifera*

**Demultiplexed sequence counts summary from QIIME2 (Bacteria)**	
Minimum Forward reads	Minimum Reverse reads
39,109	39,109
**Table summary after filtering, denoising and removal of chimeric sequences**	
Metric	Sample
Number of samples	8
Number of features	376
Total frequency	184,633
**Frequency per sample**	
	Frequency
Minimum frequency	18,691.0
1st quartile	19,669.5
Median frequency	23,032.0
3rd quartile	26,308.5
Maximum frequency	27,410.0
Mean frequency	23,079.125
**Table summary after rarefication (Bacteria)**	
Metric	Sample
Number of samples	8
Number of features	374
Total frequency	149,528
**Frequency per feature**	
	Frequency
Minimum frequency	1.0
1st quartile	5.0
Median frequency	21.5
3rd quartile	158.75
Maximum frequency	27,467.0
Mean frequency	399.807

Supplementary Table S2. Demultiplexed sequence counts and table summary of each frequency dataset and rarefaction from QIIME2 of fungi associated with *Apis mellifera*

**Demultiplexed sequence counts summary from QIIME2 (Fungi)**	
Minimum Forward reads	Minimum Reverse reads
41,252	41,252
**Table summary after filtering, denoising and removal of chimeric sequences**	
Metric	Sample
Number of samples	8
Number of features	479
Total frequency	297,731
**Frequency per sample**	
	Frequency
Minimum frequency	29,842
1st quartile	35,809.75
Median frequency	38,435.5
3rd quartile	39,738.5
Maximum frequency	40,985.0
Mean frequency	37,216.375
**Table summary after rarefication (Fungi)**	
Metric	Sample
Number of samples	8
Number of features	475
Total frequency	238,736
**Frequency per feature**	
	Frequency
Minimum frequency	1.0
1st quartile	4.0
Median frequency	10.0
3rd quartile	28.5
Maximum frequency	60,789
Mean frequency	502.602
